# Premature Ventricular Contractions From the Left Anterior Fascicle: Electrocardiographic and Electrophysiological Characteristics, Mapping Strategy, and Immediate and Long-Term Catheter Ablation Results

**DOI:** 10.3389/fcvm.2022.816237

**Published:** 2022-03-31

**Authors:** Hongwu Chen, Fangyi Xiao, Weizhu Ju, Gang Yang, Fengxiang Zhang, Kai Gu, Mingfang Li, Hailei Liu, Zidun Wang, Dinesh Sharma, Kejiang Cao, Minglong Chen

**Affiliations:** ^1^Cardiology Division, The First Affiliated Hospital of Nanjing Medical University, Nanjing, China; ^2^Department of Cardiology, The First Affiliated Hospital of Wenzhou Medical University, Wenzhou, China; ^3^Division of Cardiology, Naples Community Hospital, Naples, FL, United States

**Keywords:** left anterior fascicle, premature ventricular contraction, presystolic purkinje potential, recurrence, catheter ablation

## Abstract

**Background:**

Left anterior fascicle (LAF) premature ventricular contractions (PVC) are rarely reported. We described the electrocardiographic and electrophysiological characteristics of PVCs originating from LAF and evaluated the results of catheter ablation.

**Methods:**

The baseline AH and HV intervals were recorded during normal sinus rhythm (NSR), and the HV interval of LAF-PVC was measured during the procedure. During the index procedure, the conduction interval from the earliest Purkinje potential (PP) site to the His was labeled as time A, the conduction interval from the earliest site to the onset of the QRS as time B, then the HV interval during NSR (HV_*NSR*_) is A + B, and the HV interval during PVC (HV_*PVC*_) is B-A; a predicted PP time was calculated using HV_*NSR*_ and HV_*PVC*_. The calculated formula is as follows: Predicted target PP = (HV_*NSR*_ + HV_*PVC*_)/2. During the repeat procedure, the mapping strategy only focuses on the earliest retrograde PP due to the injury or block of LAF sustained at the index procedure.

**Results:**

Notably, 24 patients with LAF-PVC were included. The ECG characteristics of PVC exhibited right bundle branch block (RBBB) morphology with right-axis deviation (RAD) in 18 patients and only RAD in 6 patients. The QRS durations of NSR and PVC were 78.8 ± 7.9 and 106.8 ± 12.3 ms, respectively. There was no significant difference between the predicted and mapped PP site (31.5 ± 8.1 vs. 30.6 ± 7.8 ms; *P* = 0.17). There was a significant difference between the mean axis deviation before and after ablation (46.3 ± 25.4° vs. 18.3 ± 44.1°; *P* = 0.001); however, only 10 patients had a complete LAF block. Eight patients had a recurrence, the QRS morphology of LAF-PVC became narrower (95.9 ± 17.2 vs. 105.3 ± 16.9 ms, *P* = 0.003), and 4 patients’s PVC QRS morphology was similar to NSR. During the repeat procedure, the earliest retrograde PP interval was longer than the index procedure in four patients (12.0 ± 1.9 vs. 37.8 ± 1.1 ms; *P* < 0.001).

**Conclusion:**

The target PP site for ablation of the LAF region can be calculated using the HV interval during NSR and PVC at the index procedure. The mapping strategy at repeat procedures focused on the earliest retrograde PP interval.

## Introduction

Ventricular arrhythmias (VAs) arising from the left Purkinje system can be classified into left posterior fascicular, left anterior fascicular, and left upper septal (1–4). Idiopathic left ventricular tachycardia (ILVT) originating from the left posterior tachycardia is common. It can be treated with radiofrequency ablation with a high success rate, while VAs originating from the left anterior fascicle (LAF) are rare. LAF premature ventricular contractions (LAF-PVC) can be abolished by mapping using a point-to-point strategy (3) or targeting the earliest Purkinje potential (PP) (2). In this study, we investigated the following:

(1) the relationship between the predicted origin site based on our measurement and the mapped target site during LAF-PVC,
(2) electrocardiographic characteristics of LAF-PVC,
(3) the reason for recurrence and the mapping strategy during the repeat procedure.

## Materials and Methods

### Patient Population

**FIGURE 1:**
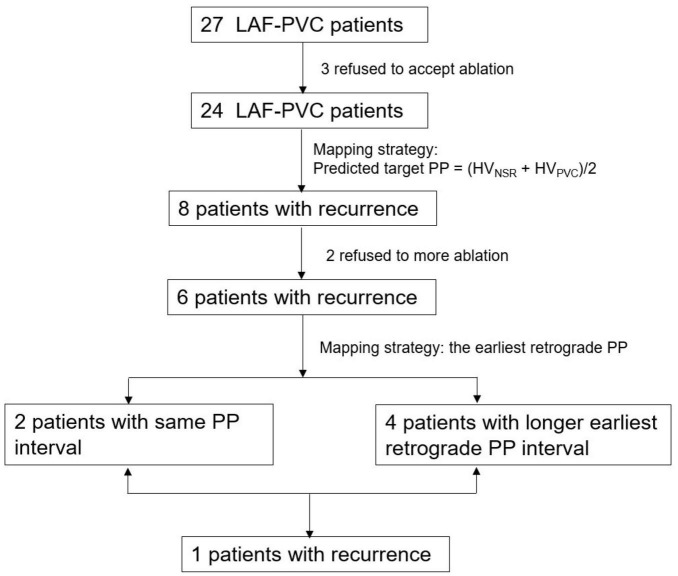
A flowchart of the study population. PVC, premature ventricular contractions; LAF, left anterior fascicle.

From January 2011 to September 2020, 27 consecutive patients (12 men, mean age 43.5 ± 17.9 years) refractory to 1.4 ± 1.0 antiarrhythmic drugs with LAF-PVC were prospectively enrolled in this study. The PVC electrocardiogram was recorded after enrollment. No patients had evidence of episodes of ventricular tachycardia. A flowchart of the study population was presented (Figure 1). The local institutional review board approved the study protocol, and all patients provided written informed consent.

### Electrophysiological Study

No patients were on amiodarone before enrollment. All antiarrhythmic drugs were discontinued for at least five half-lives before the procedure. An electrophysiological study was performed after overnight fasting in a mildly sedated state. In patients with LAF-PVC, one 6F quadripolar catheter (Diag, St. Jude Medical, Inc.) was positioned in His-bundle *via* a left femoral vein approach; a 6F decapolar catheter (Diag, St. Jude Medical, Inc.) was positioned in the coronary sinus *via* the left femoral vein for endocardial reference if an EnSite Velocity System was used. An ablation catheter guided by 3-dimensional (3-D) electroanatomic mapping (EnSite Velocity System, St Jude Medical Inc., St Paul, MN, United States; or CARTO TM, Biosense-Webster Inc., Diamond Bar, California United States) was introduced into the left ventricle (LV) *via* a retrograde aortic approach for mapping and ablation. Intracardiac electrograms were recorded using a digital electrophysiological recording system (EP-workmate Electrophysiology, St. Jude Medical, Inc., St Paul, MN, United States or LabSystem, Bard Electrophysiology, Lowell, MA, United States) and were filtered from 30 to 300 Hz.

The baseline AH and HV intervals were recorded during normal sinus rhythm (NSR). In patients with LAF-PVC, the HV and coupled interval during PVC were measured. If LAF-PVC did not occur at baseline, then isoproterenol infusion (1–4 μg/min) was given to increase the heart rate by 30%. If the LAF-PVC was non-inducible, the case was excluded.

According to previous studies, the origin of LAF-PVC is located in the LAF at the site of the earliest retrograde PP (1, 2, 5), and the myocardial breakthrough site is adjacent to the LAF during PVC (5). The mapping strategy during the index procedure developed from our center has been previously described (1). In brief, the conduction interval from the earliest PP site to His was labeled as time A, the conduction interval from the earliest site to the onset of the QRS was delineated as time B, the HV interval during NSR (HV_*NSR*_) was A + B, the HV interval during PVC (HV_*PVC*_) was B-A, and the predicted target PP (B) was calculated from the HV_*NSR*_ and HV_*PVC*_. The calculated formula is as follows: Predicted target PP = (HV_*NSR*_ + HV_*PVC*_)/2. During the repeat procedure, the mapping strategy focused on the earliest retrograde PP due to the injury or block of LAF.

### Mapping and Ablation

Left ventricle geometry and activation mapping *via* a retrograde approach were created by 3-D mapping (CARTO system, Biosense-Webster Inc., Diamond Bar, CA; or EnSite Velocity System, St Jude Medical Inc., St Paul, MN, United States). Activation mapping during PVC was performed along the LAF to identify the earliest PP by 3D activation mapping. As a result, radiofrequency ablation energy utilizing a 4-mm non-irrigated catheter was delivered at the earliest PP during PVC. The power output was titrated to 30 W with a maximum target temperature of 50–60°C for 60–120 s. If the origin site was closer to the distal left bundle branch, the power output was started at 10 W and titrated to 20 W to avoid left bundle branch injury. Acute procedural success was defined as the termination of the LAF-PVC during ablation and the absence of arrhythmias within 30 min of the final radiofrequency ablation application despite programmed electrical stimulation with isoproterenol infusion.

### Follow-Up

Surface ECGs and 24-h Holter recordings were performed regularly following the procedure and then at 3 and 6 months after discharge. Furthermore, an ECG was performed anytime if patients experienced palpitations.

### Statistical Analysis

Data were analyzed using SPSS 18.0. Continuous variables are expressed as the mean ± SD. Then, the Mann-Whitney two-sample test examined group differences for continuous data. The categorical variables were compared with the chi-square test. A *P*-*value* of <0.05 was considered statistically significant.

## Results

### Patient Characteristics

Among the 27 patients, three patients were excluded because two patients were non-inducible, and one patient had common iliac artery dissection while introducing an ablation catheter to the LV. The remaining 24 patients (10 men, mean age 40.7 ± 17.2 years) were enrolled in this study. The mean PVC burden and coupled interval were 18.8 ± 7.7% and 503.7 ± 63.1 ms, respectively. Their mean left ventricular ejection fraction and left ventricular diastolic diameter were 64.1 ± 3.2% and 46.0 ± 3.9 mm, respectively.

All patients had clinical palpitations for a mean of 39.9 ± 43.7 months. Eleven patients had no history of using verapamil, and only six of the remaining patients (46%) were sensitive to verapamil. The ECG characteristics of PVC exhibited right bundle branch block (RBBB) morphology with right axis deviation (RAD) in 18 patients and only RAD in 6 patients (Table 1); the mean axis deviation was 46.3 ± 25.4°. The QRS pattern of the LAF-PVC was roughly shaped like qR in leads II, III, and aVF. Interestingly, the QRS pattern of the LAF-PVC in V_1_ was demonstrated as rs (2 cases), rS (2 cases), Rs (2 cases), rsr′ (2 cases), and rsR′ (16 cases). The QRS durations of NSR and PVC were 78.8 ± 7.9 and 106.8 ± 12.3 ms, respectively. The mean ratio of QRS duration with NSR and PVC was 0.74 ± 0.06.

**TABLE 1:** Baseline characteristics of patients with premature ventricular contractions originated from the left anterior fascicle.

No.	Sex	Age (years)	Diagnosis	History (M)	PVC burden (%)	Morphology	LVDd	LVEF (%)	Numbers of AAD (N)	Verapamil	Follow up (M)
1	M	21	LAF-PVC	12	17.8	RAD	49	63.7	1	+	96
2	F	31	LAF-PVC	24	20.0	RBBB/RAD	48	62.1	1	–	87
3	M	50	LAF-PVC	12	19.8	RBBB/RAD	40	63	1	NO	77
4	F	15	LAF-PVC	120	20.5	RBBB/RAD	41	69.9	0	NO	77
5	F	57	LAF-PVC	120	11.0	RAD	42	65	1	NO	65
6	F	46	LAF-PVC	14	17.0	RBBB/RAD	43	62.1	3	NO	63
7	M	48	LAF-PVC	46	13.6	RBBB/RAD	40	66.2	1	NO	62
8	F	38	LAF-PVC	24	22.9	RAD	49	60	1	+	51
9	M	24	LAF-PVC	24	20.6	RBBB/RAD	40	62.1	1	–	49
10	F	23	LAF-PVC	12	14.4	RBBB/RAD	46	64	1	NO	49
11	F	40	LAF-PVC	60	19.9	RBBB/RAD	47	68	2	NO	48
12	F	51	LAF-PVC	4	12.4	RBBB/RAD	50	70.4	0	NO	46
13	M	71	LAF-PVC	180	49.0	RAD	46	66.9	2	+	42
14	M	19	LAF-PVC	12	12.6	RBBB/RAD	49	63.7	0	–	40
15	M	27	LAF-PVC	4	12.5	RBBB/RAD	45	59	3	+	37
16	M	74	LAF-PVC	24	14.8	RAD	48	67.4	4	+	37
17	F	38	LAF-PVC	6	12.8	RBBB/RAD	44	62.1	1	NO	25
18	F	37	LAF-PVC	36	12.7	RBBB/RAD	49	60.1	1	NO	23
19	F	38	LAF-PVC	3	22.0	RBBB/RAD	41	60.2	0	NO	19
20	M	75	LAF-PVC	60	28.8	RBBB/RAD	54	62.1	2	+	18
21	F	59	LAF-PVC	84	22.1	RBBB/RAD	49	63.7	1	–	17
22	M	20	LAF-PVC	24	12.6	RAD	49	69	1	–	16
23	F	46	LAF-PVC	24	18.2	RBBB/RAD	43	61.2	2	–	15
24	F	29	LAF-PVC	29	22.9	RBBB/RAD	52	65.9	3	–	3
Mean		40.7		39.9	18.8		46.0	64.1	1.4		44.2
STD		17.2		43.7	7.7		3.9	3.2	1.0		24.8

*AAD, anti-arrhythmic drugs; LAF, left anterior fascicle; LVDd, left ventricle diastolic diameter; LVEF, left ventricular ejection fraction; M, months; N, number; PVC, premature ventricular contractions; NA, no available; RAD, right axis deviation; RBBB, right bundle branch block; +, sensitivity to verapamil; –, not sensitivity to verapamil.*

### Mapping and Ablation Left Anterior Fascicle-Premature Ventricular Contractions During the Index Ablation Procedure

**FIGURE 2:**
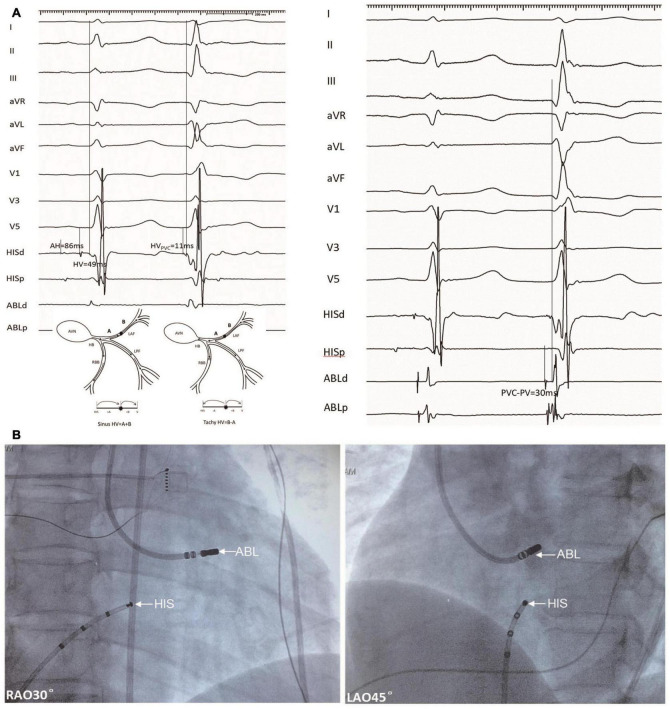
An example of a target ablation site for a PVC originating from the LAF (Patient 18). **(A)** Left panel, HV intervals during NSR and PVC with mapping electrode positioned at the His bundle were 49 and 11 ms, respectively. Right panel, the catheter was positioned at the target site with a recording interval of 30 ms during PVC between local fascicular potential (PP) and surface ECG, this is equal to the predicted value. **(B)** Fluoroscopy of LAF-PVC target. PVC, premature ventricular contractions; LAF, left anterior fascicle; NSR, normal sinus rhythm; ABL, ablation electrode; H-V, His bundle to the onset of surface ventricular activation; RAO, right anterior oblique; and LAO, left anterior oblique.

According to the inferior leads of ECG, all PVC patients had an origin from the LAF. The mean AH, HV interval during NSR, and HV interval during LAF-PVC were 88.6 ± 14.5 ms, 47.7 ± 6.0 ms, and 15.7 ± 12.4 ms, respectively (Table 2). There was no significant difference between the predicted and mapped values (31.5 ± 8.1 vs. 30.6 ± 8.0 ms; *P* = 0.17), and three patients had differences in predicted and mapped values due to unstable ABL catheters. The acute success rate was 100% during the procedure. Eight patients had a recurrence, six of whom recurred at an average of 1.3 ± 0.5 days after the index procedure. Three patients with different predicted and mapped values had a recurrence of PVC (Table 2); five patients with the same values had a recurrence. All patients without recurrence had the same predicted and mapped earliest PP interval, as illustrated in Figure 2. In patients with recurrence, the QRS morphology of NSR had slight changes; however, the QRS morphology of PVC became narrower than the basic pattern (95.9 ± 17.2 vs. 105.3 ± 16.9, *P* = 0.003), and four patients had a similar QRS morphology of PVC compared with NSR. There was a significant difference in the mean axis deviation before and after ablation during NSR (46.3 ± 25.4° vs. 18.3 ± 44.1°; *P* = 0.001); however, only ten patients had a complete LAF block. Furthermore, there was no significant difference between the mean axis deviation in the successful and unsuccessful groups after the index ablation procedure (16.7 ± 46.7° vs. 32.3 ± 31.7°; *P* = 0.45).

**TABLE 2:** Electrophysiological characteristics and follow-up result after the index ablation procedure.

No.	Age	NSR QRS duration	PVC QRS duration	NSR/PVC QRS ratio	PVC pattern*	Coupled interval (ms)	Axis before ablation	Axis after ablation	AH-SR (ms)	HV-SR (ms)	HV-PVC (ms)	Predicted PP site (ms)	Mapped PP site (ms)	NSR PP site (ms)	LAF block	Success
1	21	80	100	0.80	qR/rs	560	67	11	96	53	38	45	45	45	+	+
2	31	80	120	0.67	qR/rsR′	460	−8	−13	90	42	12	27	27	27	–	+
3	50	90	120	0.75	qR/rsR′	464	55	64	84	52	24	38	38	38	–	+
4	15	65	98	0.66	qR/rsR′	564	63	53	75	45	15	30	30	30	–	–
5	57	80	118	0.68	qR/Rs	544	45	58	100	56	34	45	32	32	+	–
6	46	80	120	0.67	qR/rsR′	480	46	NA	92	55	6	30	20	20	+	–
7	48	104	132	0.79	qR/rsR′	520	48	50	100	35	25	30	30	30	–	+
8	38	80	106	0.75	qR/rs	620	21	30	96	46	12	29	29	29	+	–
9	24	82	100	0.82	qR/rsR′	520	21	11	110	46	22	34	34	34	–	+
10	23	63	82	0.77	qR/rsR′	596	73	68	100	50	25	37	40	40	+	–
11	40	84	106	0.79	qR/rsR′	596	43	−7	106	50	0	25	25	25	–	–
12	51	76	96	0.79	qR/rsR′	428	73	63	84	52	16	34	34	34	–	+
13	71	82	106	0.77	qR/rS	528	67	−7	66	50	42	46	46	46	+	+
14	19	80	106	0.75	qR/rsR′	426	30	11	116	54	0	27	27	27	–	+
15	27	74	96	0.77	qR/rsR′	460	81	80	100	40	20	30	30	30	+	+
16	74	80	108	0.74	qR/Rs	484	−18	−90	68	46	10	28	28	28	–	+
17	38	72	88	0.82	qR/rsR′	484	82	53	66	54	12	33	33	33	+	+
18	37	70	90	0.78	qR/rsR′	440	60	54	80	49	11	30	30	30	–	–
19	38	80	118	0.68	qR/rsR′	545	60	60	80	48	20	34	34	34	+	+
20	75	80	110	0.73	qR/rsR′	480	30	−42	108	52	26	39	39	39	+	+
21	59	78	128	0.61	qR/rsR′	404	47	−22	68	38	−8	10	10	10	–	+
22	20	72	96	0.75	qR/rs	440	53	−30	74	54	10	32	32	32	–	–
23	46	76	110	0.69	qR/rsR′	612	15	21	80	40	5	22	22	22	–	+
24	29	82	108	0.76	qR/rsR′	434	56	−49	87	40	0	20	20	20	–	+
Mean	40.7	78.8	106.8	0.74		503.7	46.3	18.3	88.6	47.7	15.7	31.5	30.6	30.6		
STD	17.2	7.9	12.3	0.06		63.1	25.4	44.1	14.5	6.0	12.4	8.1	8.0	8.0		

*LAF, left anterior fascicle; NA, no available; PVC, premature ventricular complex; NSR, normal sinus rhythm; TCL, tachycardia cycle length.*

*The QRS pattern of PVC in leads II, III, and avF and the lead V1, respectively.

### Mapping and Ablation Left Anterior Fascicle-Premature Ventricular Contractions During the Second Ablation Procedure

Eight patients who had documented recurrence of LAF-PVC were recommended to undergo a repeated ablation procedure. Six patients consented to the second ablation. All eight patients had a narrower LAF-PVC QRS duration compared with the index procedure (102.0 ± 13.1 vs. 83.6 ± 8.4; *P* = 0.0003), and five patients had the change of LAF-PVC QRS morphology in lead V_1_, three patients PVC-QRS morphology changed from rsR′ to rS, one from Rs to rS, one from Rs to rs; the remaining three patients had the similar morphology.

**FIGURE 3:**
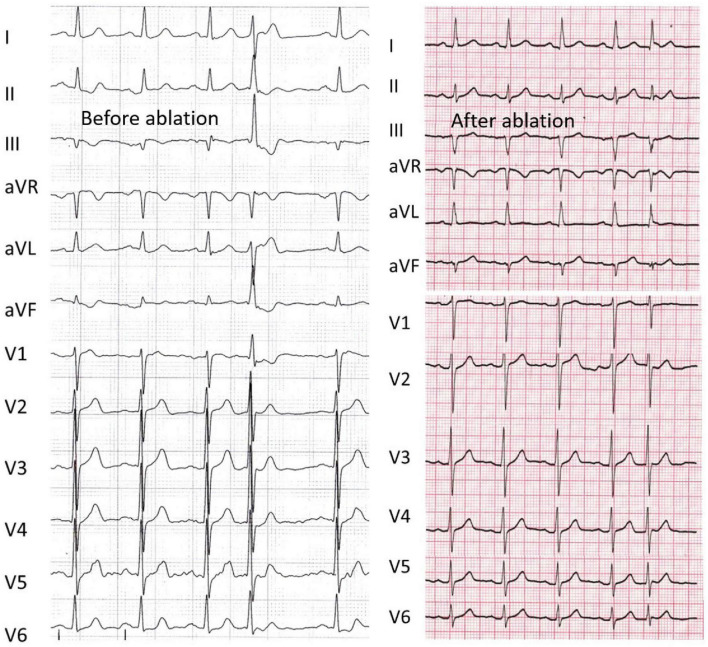
An example of a failed case that refused more procedures (Patient 5). Left panel, the axis deviation was 45° before ablation, and radiofrequency energy was delivered at the distal site. Right panel, the patient had recurrence after the index procedure, the QRS duration ratio of NSR/PVC after ablation increased from 0.68 to 0.93, and the axis deviation increased to 58°. The abbreviations are as in Figure 2.

Figure 3 shows an example of the failed case in patient 5, who refused repeat ablation. During the first procedure, the AH_*NSR*_, HV_*NSR*_, and HV_*PVC*_ intervals were 91, 56, and 34 ms, respectively; the axis deviation was 45°. The predicted value was 45 ms, and the earliest PP interval was also mapped with 45 ms; however, the radiofrequency energy was delivered at the distal site (32 ms), the QRS duration ratio of NSR/PVC after ablation increased from 0.68 to 0.93, and the axis deviation increased to 58°.

**FIGURE 4:**
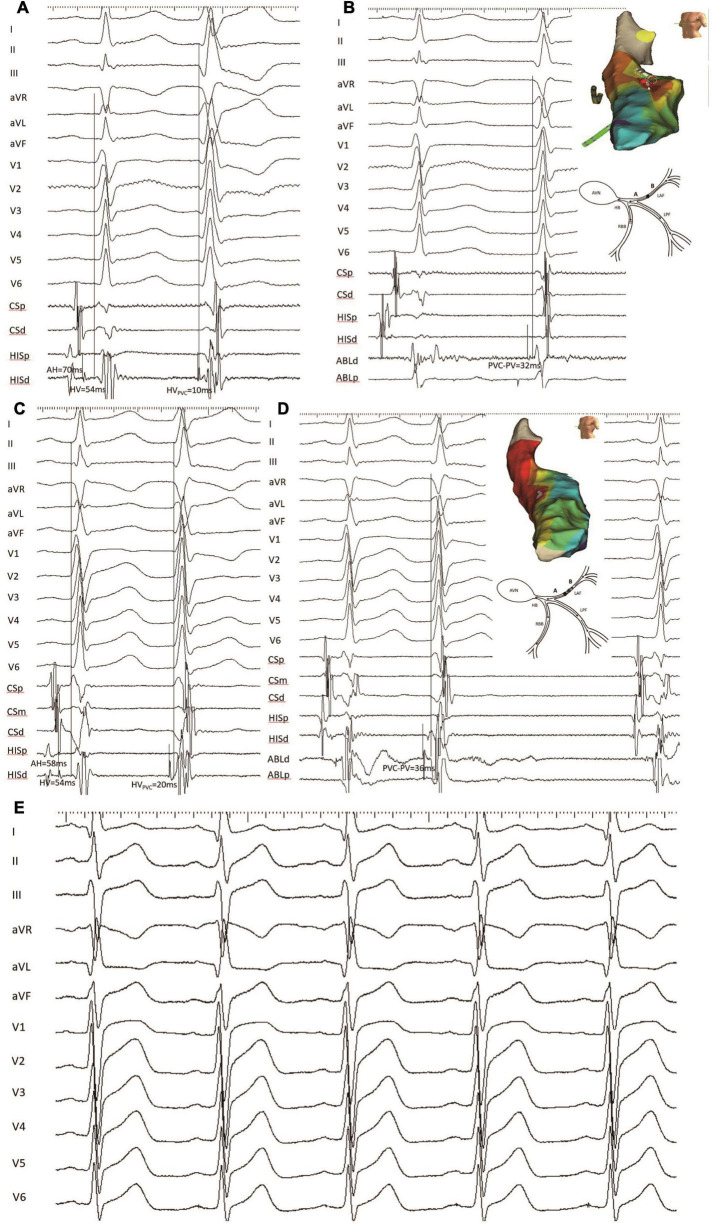
An example of a failed patient with LAF-PVC who underwent a repeat ablation procedure (patient 22). **(A)** HV interval during NSR and PVC with mapping electrode positioned at the His bundle was 54 and 10 ms, respectively. **(B)** The target site was mapped with recordings of local fascicular potential-the onset of the QRS interval of 32 ms during PVC. Three-dimensional activation mapping of the left anterior area showed the target site, and a schematic diagram showed conduction within the Purkinje system. **(C)** During the repeat procedure, the HV_*NSR*_ interval was the same as that during the first procedure, while the HV_*PVC*_ interval was 20 ms; the QRS duration after ablation decreased from 96 to 80 ms, and the axis deviation increased to 21°. Note that NSR-QRS morphology had a slight change, and the QRS duration of PVC was slightly narrower than that before ablation. **(D)** The same site was identified, and the earliest retrograde PP with 36 ms was mapped and had successful ablation. Three-dimensional activation mapping of the left anterior area showed the target site and a schematic diagram showing the slow conduction within the LAF fiber. It is noted that the target PP was sharp during PVC, while the PP was fragmented during NSR. **(E)** The electrocardiogram showed the left anterior block after successful ablation. CS, coronary sinus; PP, Purkinje potential; the other abbreviations are as in Figure 2.

**FIGURE 5:**
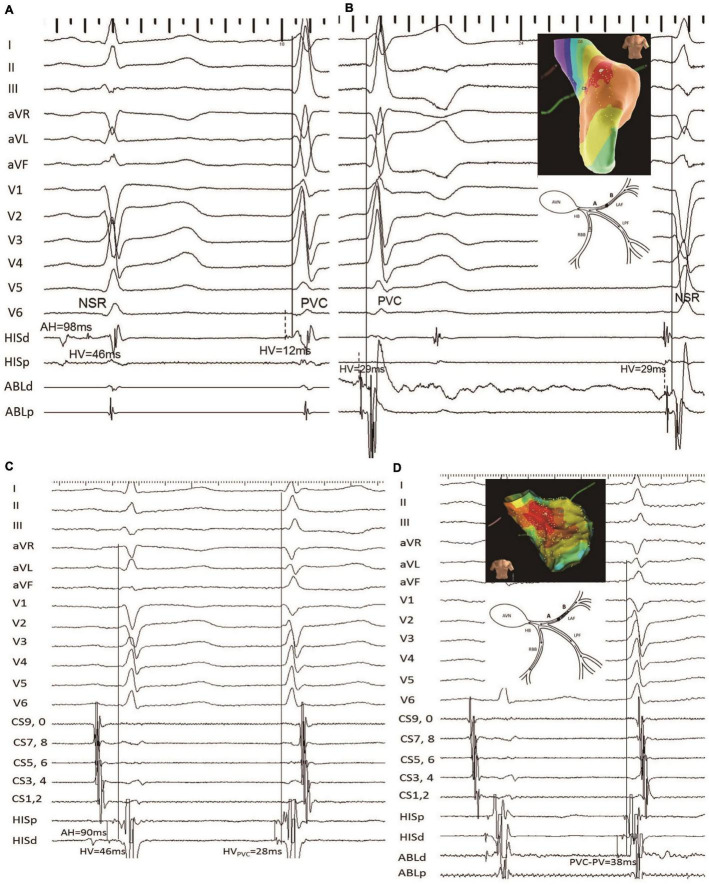
Another example of failed LAF-PVC case (patient 8). **(A)** The HV intervals during SNR and PVC with the mapping electrode positioned at the His bundle were 46 and 12, respectively; it is noted that the predicted value was 29 ms. **(B)** The catheter was positioned at the target site with recordings of local anterior PP-the onset of surface ECG interval of 29 ms during PVC. Three-dimensional activation mapping of the left anterior area shows the target site. **(C)** The HV_*NSR*_ interval was the same as that of the first procedure, while the HV_*PVC*_ interval was 28 ms; the QRS duration ratio of NSR/PVC increased from 0.75 to 0.95; and the QRS morphology of PVC was similar to the NSR-QRS morphology. **(D)** The earliest retrograde PP with 38 ms was mapped and had successful ablation. Three-dimensional activation mapping of the left anterior area showed the target site and a schematic diagram showing the slow conduction within the LAF fiber, which might explain the reason for the narrower QRS. It is noted that the target PP was fragmented during PVC. CS, coronary sinus; the other abbreviations are as in Figure 2.

During the repeat procedure of the six consented patients, the earliest retrograde PP interval during PVC was mapped. Four patients had different predicted and mapped values. The earliest retrograde PP interval during PVC was mapped to the vicinity of the LAF, to a similarly located site of the index procedure in four cases. The earliest retrograde PP interval was longer than the index procedure (12.0 ± 1.9 vs. 37.8 ± 1.1; *P* < 0.001) (Figures 4, 5).

Figure 4 (patient 22) shows that the HV_*NSR*_ interval was the same as that of the first procedure, while the HV_*PVC*_ interval was 20 ms; the QRS duration after ablation decreased from 96 to 80 ms; the PVC morphology was similar to baseline. The NSR-QRS morphology slightly changed; the same site was identified, and the earliest retrograde PP with 36 ms was mapped and had successful ablation using 3-D activation mapping. A schematic diagram demonstrates slow conduction within the LAF fiber, and the electrocardiogram reveals a left anterior block after successful ablation.

Figure 5 demonstrates that in patient 8, the HV_*NSR*_ interval was the same as that of the first procedure, while the HV_*PVC*_ interval was 28 ms. The QRS duration ratio of NSR/PVC increased from 0.75 to 0.95, and the PVC morphology was similar to the NSR-QRS morphology. The catheter was positioned at the same site with recordings of local anterior PP-the onset of a surface ECG interval of 38 ms during PVC compared with the index procedure.

### Final Follow-Up Results

After a mean follow-up of 44.3 ± 24.8 months after the last ablation procedure, three patients had a recurrence and were prescribed metoprolol to control symptoms. All other patients had no arrhythmia recurrence.

### Complications

No complications were observed in the study patients during the perioperative period.

## Discussion

### Major Finding

This is the first report of the long-term follow-up results of LAF-PVC in a relatively large sample. Our study found that the target site can be calculated by the HV interval during NSR and PVC. Locations of recurrent PVCs in repeat procedures were anatomically close to the ablation sites during the index procedure even though the QRS morphology of the PVC is different compared with the index procedure. The QRS morphology of LAF-PVC is dependent on how slow conduction over LAF occurs in recurrence patients. If the conduction velocity is slightly slower over LAF, the PVC morphology might be similar, but the QRS duration becomes narrower; if the conduction velocity is rebalanced over both left fascicles, the PVC-QRS morphology might be similar to NSR.

### The Anatomy of the Left Anterior Purkinje System

Basic histological studies or postmortem examination have demonstrated that the left His-Purkinje system is a fan-like structure with a bifascicular structure or three major divisions; which means left posterior fascicle (LPF) and LAF with or without left middle fascicle (6–8). Long et al. elucidated the entire left His-Purkinje system in the human heart during NSR (5). Their study demonstrated that 92% of patients bifurcated into LAF and LPF divisions. Furthermore, the length of LAF was shorter than that of LPF. They also identified that the activation time of LAF was shorter than that of LPF in 24 patients, who presented preferential conduction over LAF with a single ventricular breakthrough site.

### The Mechanism of Different QRS Morphology in Recurrent Patients

Left ventricular arrhythmia originating from the left posterior fascicle is common. The QRS morphology shows RBBB and left axis deviation (9). Ventricular arrhythmia with QRS morphology similar to that of NSR or incomplete RBBB was demonstrated to arise from the left upper septum (4). In our study, all patients shared small q waves and tall R waves in inferior leads, and most of the patients (74%) showed incomplete RBBB in the V_1_ lead. The mechanism might be preferential conduction over LAF. In patients with proximal LAF origin, the QRS morphology of V_1_ was similar to that of the NSR electrocardiogram at baseline. In the patients with recurrence, the PVC-QRS duration was narrower (102.0 ± 13.1 vs. 83.6 ± 8.4; *P* = 0.0003), and five patients had a change in LAF-PVC QRS morphology in lead V_1_. Three patients had PVC-QRS morphology that changed from rsR′ to rS, one from Rs to rS, and one from Rs to rs. The similarity between PVC-QRS morphology during the index and repeat procedure was identified in three patients. This phenomenon might be explained as follows:

1. Ineffective ablation lesions.
2. If there was slower conduction, which means the preferential conduction was still over LAF than LPF, the PVC-QRS duration was slightly narrower with a similar morphology after the index ablation.
3. If there was further slower conduction over LAF, which led to rebalancing conduction over both left fascicles, the PVC-QRS duration was narrower, and the PVC morphology was similar to that of NSR.
4. If there was block conduction over LAF, we can speculate that the PVC-QRS morphology might show left axis deviation.

Previous studies have demonstrated changes in QRS morphology in recurrent patients with idiopathic left posterior ventricular tachycardia. Zhang et al. recently reported that upper septal ventricular tachycardia is identified in recurrent patients with the previous ablation of typical ILVT, which led to narrow QRS morphology (10). Other studies also had similar findings (4, 11). Their mechanism of recurrence is reentry or a new focal origin. However, in our study, the recurrent patients had the same origin. The change in QRS morphology depends on the conduction velocity over LAF, different from previous studies (4, 10, 11).

### The Mapping and Ablation Strategy for Left Anterior Fascicle-Premature Ventricular Contractions

During the index procedure, a previous study showed a simple point-to-point mapping strategy along with the left anterior fiber in two cases. If the earliest activation time was mapped, the target site was identified. They also found that the target site’s fascicular potential to ventricle interval is identical during NSR and PVC (3). Recently, Wang et al. (2). reported a selective method in three cases. They found that the target site should be identified by the earliest presystolic PP but not the earliest ventricular activation mapping in patients with LAF-PVC. Our previous study (1) showed a straightforward method with a formula to identify the target site in a series of patients with ILVT. The calculated formula is as follows: Predicted target PP = (HV_*NSR*_ + HV_*PVC*_)/2. This study also proves this formula in patients with LAF-PVC, and the success rate was 76% (16/21). All three cases had recurrence with different predicted and mapped values during the index procedure and the repeat procedure (3/3, 100%). One of the cases had recurrence with narrower PVC-QRS morphology (Figure 3). Patients with the same predicted and mapped values who had recurrence might have poor contact.

The earliest PP is suggested in previous studies during the repeat procedure with recurrence LAF-PVC cases (10, 11). However, no study has focused on LAF-PVC or mentioned the reasons for recurrence. Our study is the first to describe the mapping strategy with the earliest retrograde PP in a series cohort of patients with LAF-PVC. This method is simple but effective for identifying the target site. The QRS morphology depends on the LAF conduction characteristics; our study showed that PVC-QRS could be similar to NSR during the repeat procedure despite the same LAF site of origin, not near the His area. However, if we focused on the His region according to narrower PVC-QRS morphology, an unnecessary third-degree atrioventricular block might occur, and the LAF-PVC would also recur. In this study, the earliest retrograde PP was mapped in the LAF region in recurrent cases, and the success rate was 83% (5/6), even though the PVC-QRS morphology differed.

The ablation endpoint in ILVT was controversial. Some studies demonstrated that a complete LPF block is associated with a high procedural success rate (12, 13); others showed that LPF block is not necessary for success (14, 15). In patients with LAF-PVC, controversy also exists. A previous study demonstrating changes in the QRS axis and morphology noted in two cases indicated proximal LAF block (3). In our study, there was a significant difference in the mean axis deviation before and after ablation (46.3 ± 25.4° vs. 18.3 ± 44.1°; *P* = 0.002). However, LAF block was only identified in ten cases (41.7%). Therefore, we can speculate that the LAF block was unnecessary to predict success even though there were changes in the QRS axis or morphology. The LAF block was just a byproduct following LAF region ablation.

### Limitations

First, this study involved only a small number of patients due to rare cases of LAF-PVC. Second, we used the formula to predict the origin site; however, the ventricular breakthrough site may affect the HV interval during NSR, which could lead to incorrect calculation of the predicted location. Third, we did not analyze the PVC mechanism; and fourth, verapamil was prescribed in 13 patients, and verapamil sensitivity was only identified in six patients.

## Conclusion

The target PP site for ablation of the LAF region can be calculated using the HV interval during NSR and PVC. During the repeat procedures, the earliest retrograde PP should be targeted.

## Data Availability Statement

The original contributions presented in the study are included in the article/supplementary material, further inquiries can be directed to the corresponding author.

## Ethics Statement

The studies involving human participants were reviewed and approved by the local institutional review board. Written informed consent to participate in this study was provided by the participants’ legal guardian/next of kin.

## Author Contributions

MC and HC participated in the design of this study. HC, FX, WJ, GY, and FZ mapped and ablated all the cases. ML, KG, ZW, and HL collected and performed the statistical analysis. HC and FX wrote the first draft of the manuscript. DS revised the manuscript. MC and KC monitored this study. All authors provided input on data analysis and interpretations, participated in multiple revisions of the manuscript, approved the final version of the manuscript, and agreed to be accountable for all aspects of the work.

## Conflict of Interest

The authors declare that the research was conducted in the absence of any commercial or financial relationships that could be construed as a potential conflict of interest.

## Publisher’s Note

All claims expressed in this article are solely those of the authors and do not necessarily represent those of their affiliated organizations, or those of the publisher, the editors and the reviewers. Any product that may be evaluated in this article, or claim that may be made by its manufacturer, is not guaranteed or endorsed by the publisher.

## Abbreviations

ECG: electrocardiogram
ILVT: idiopathic left ventricular tachycardia
LAF: left anterior fascicle
LAF-PVC: LAF premature ventricular contractions
LPF: left posterior fascicle
LV: left ventricle
NSR: normal sinus rhythm
PP: Purkinje potential
PVC: premature ventricular contractions
RAD: right axis deviation
RBBB: right bundle branch block
VAs: ventricular arrhythmias.
